# Supplementary material for: Identification of the Telomere elongation Mutation in Drosophila
Source: Cells. 2022 Nov 3;11(21):3484. doi: 10.3390/cells11213484 (PMC9659042; doi:10.3390/cells11213484)
Supplement: Supplementary file 1 [file cells-11-03484-s001.zip › cells-1930486-supplementary.pdf]

Identification of the *Telomere elongation* mutation in *Drosophila*

Hemakumar M. Reddy, Thomas A. Randall, Francesca Cipressa, Antonella Porrazzo, Giovanni Cenci, Radmila Capkova Frydrychova and James M. Mason

Supplementary Figures and Text

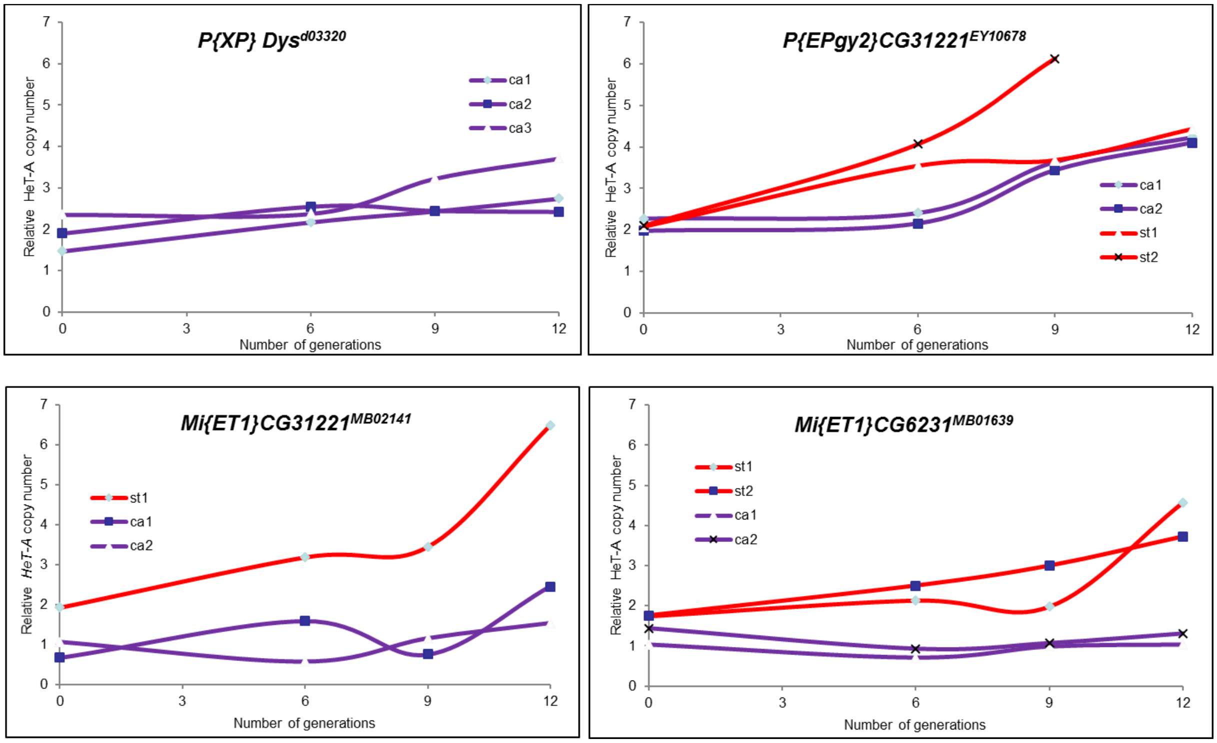

Figure S1 Telomere length in recombinants from transposons used for *Tel*^{1} mapping. Change in relative *HeT-A* copy number in different recombinant stocks from generation 0 to 12. Recombinants carrying *ca* are shown in purple; those bearing *st* in red.

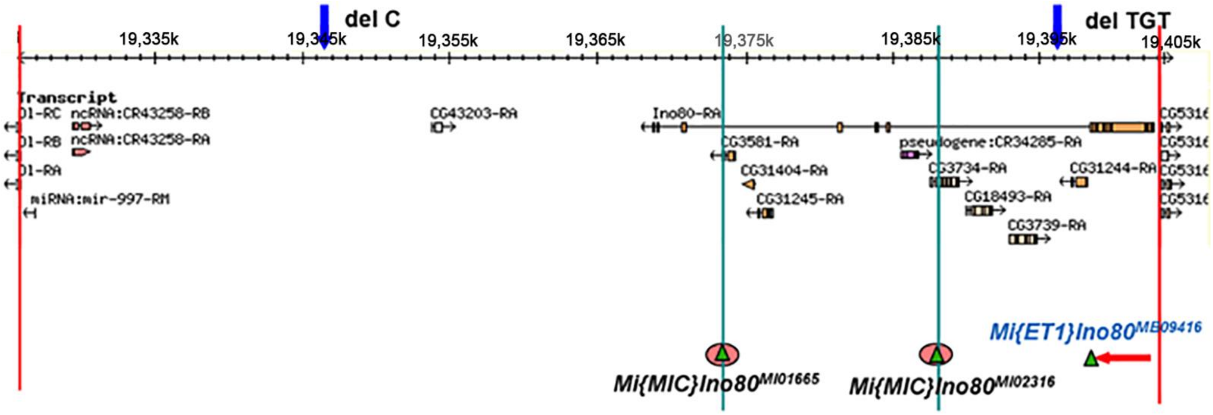

Figure S2 FlyBase browser map of the 77kb *Tel*[1] region with indels and *Minos* insertion sites. Genome browser map for the 77 kb region between *05151* and *d10097* (red vertical lines). Blue arrows show the location of two candidate indels (deletion C and deletion TGT). Two transposon insertions, *MI03112* and *MI02316*, which lie between the two indels, were used for further mapping the *Tel*[1] mutation. Red arrow indicates the *MB09416* transposon insertion site. Flies carrying this insertion have long telomeres.

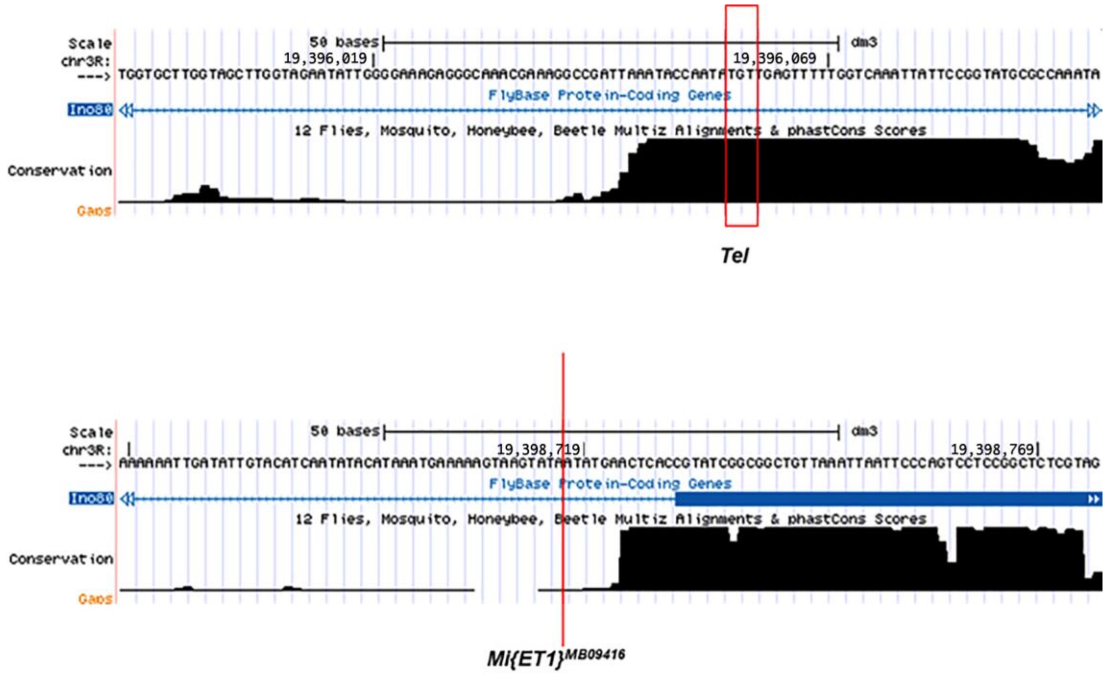

Figure S3 Sequence conservation among insect species at *Tel*[1] and *MB09416* insertion loci. UCSC Genome Browser maps showing conservation in the immediate vicinity of the *Tel*[1] mutation (deletion TGT) and the *MB09416* insertion. The sequence around the *Tel*[1] mutation shows good conservation among different Drosophila and insect species, whereas the sequence surrounding the *MB09416* insertion site is not well conserved.

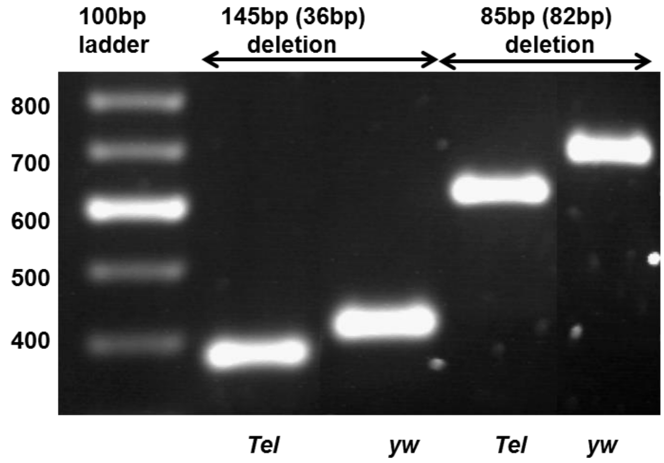

Figure S4. PCR with flanking primers to large indels found in *Tel* genome. PCR products spanning two large indels 145 bp (3R: 19,331,416 – 560 and 85 bp (3R:19,344,721-800) from *Tel* and *y w*. These indels were identified in the *Tel*^{1} genome by manual scanning the CLC Genomics assembly. PCR product of 145 bp deletion shows a band size difference of ~40 bp, whereas 85 bp deletion shows expected band size variation.

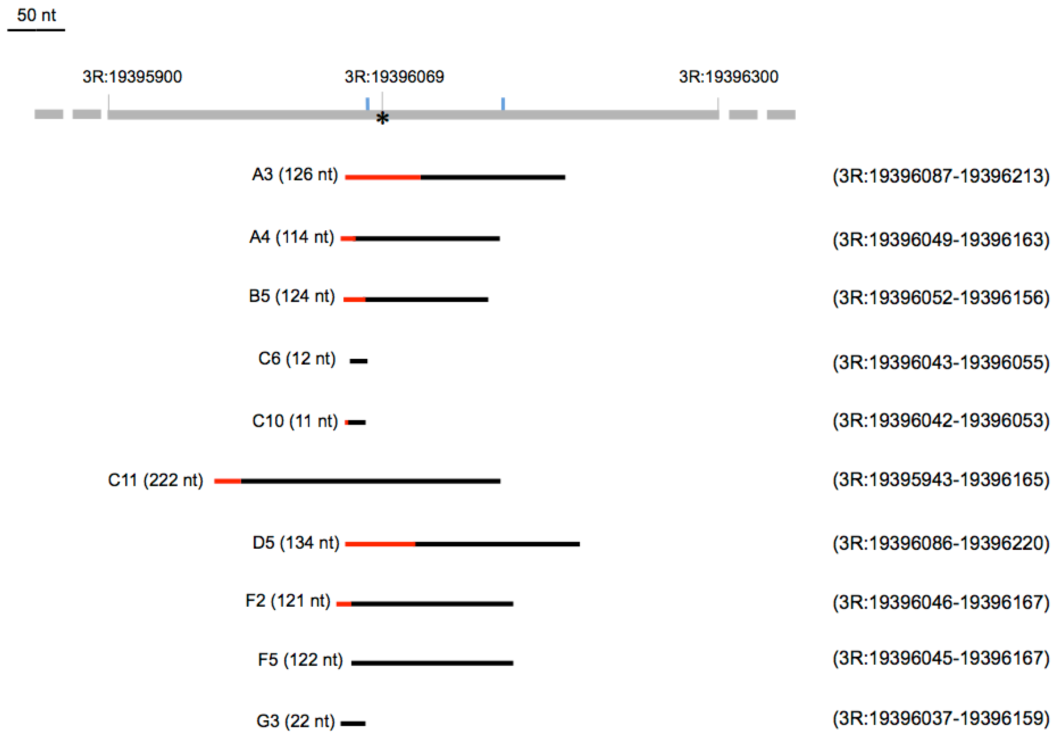

Figure S5. **Schematic map of CRISPR/Cas 9-induced deletions**. Black bars indicate the extension of the deletion with respect to the *dIno80* intron region (gray bar on the top). The size in nt of each deletion is shown in parenthesis while the corresponding molecular coordinates on chromosome 3R are indicated on the right. Red bars represent the extension of "spurious" sequences found at the 5' breakpoint. Blue vertical lines show the position of Crispr/Cas9 induced breaks. The asterisk indicates the position of the TGT 3nt sequence. Gray vertical bars refers to molecular coordinates of the *dIno80* region

Supplementary Methods

Genome sequencing Genome sequencing was done on an Illumina GA IIx sequencer. Five micrograms of genomic DNA was sheared to ~400 bp using a Covaris E210 with settings: duty cycle 20%, intensity 4, cycles 200. The DNA was size-selected for the range 300-600 bp on a 2% agarose gel and extracted using a Gel Extraction Kit (Qiagen). The library was constructed using NEB Next DNA Library Prep Master Mix Set for Illumina (New England Biolabs) and Illumina's Paired-End DNA Sample Prep Oligo Only Kit. The resulting library was selected on a 2% agarose gel for 440-560 bp and extracted using a Gel Extraction Kit (Qiagen). To insure that the library was not over-amplified a test amplification was performed in which aliquots of a PCR reaction were removed every 2 cycles from 4-16. These aliquots were evaluated on a 2% agarose gel and an optimal cycle number was selected for subsequent large scale amplification in which unique barcodes were added to each library. Eight cycles were selected for these libraries. The amplification reaction was cleaned using two rounds of AMPure XP Beads (Agencourt). Each library was run in one lane of a GAIIx to generate paired-end, 101 base reads. Data was processed using RTA1.6.32.0 and GERALD 1.15. For *Tel* 66,654,840 reads were obtained and for *y w* 76,757,736 reads, representing genome coverage of 48X and 55X, respectively.

4174276

Supplementary Results

Analysis of large indels Large indels (>5 bp) identified by manually scanning the *Tel*^{1} genome assembly in the 79 kb region (3R: 19,325,276-19,404,276). To confirm the identity of these indels, we designed primers flanking these indels, which were then PCR amplified and sequenced by Sanger sequencing. Figure S4 shows the 145 bp (3R: 19,331,416 – 560) and 85 bp (3R: 19,344,721-800) indels initially identified in *Tel*^{1} but not *y w*. Sequencing of these PCR fragments showed that; the 145 bp 'deficiency' is actually many small deletions (6 bp, 5 bp, 23 bp and 2 bp) present very close to each other; the 85 bp deficiency is actually a 82 bp deletion in Tel; the 20 bp insertion (3R: 19,337,636) is actually three closely linked insertions (1 bp, 11 bp and 7 bp). All these indels were also found in one or more of DGRP lines, so they are all natural polymorphisms.

The CLC generated assembly suggested a 38 bp deletion at 3R:19,371,152 in both *Tel* and *y w*. This region is part of exon11 of *Ino80* coding region and thus of potential functional importance. To confirm the presence/absence of this deletion we PCR amplified this region and re-sequenced by Sanger sequencing. This showed complete identity to the reference sequence in both the Tel1 and y w genomes at this region, suggesting the apparent deletion was an assembly artifact, possibly due to the high GC content in this region.

Sequence Analysis of CrispR/Cas9-induced deletions in the Ino80 intron 8 showing the extension of the deletions.

“Clean” Breaks

Sequence of deletion-bearing lines which contain intact junction sequences at the 5’breakpoint (sequence in bold). The TGT sequence is shown in red. For each sequence, the extension of deletion in nt and the molecular coordinates are provided

C6>
12nt deletion (3R:19396043-19396055)

ATGATCTGCGGAGGAAGAGCGGAGATCCTACACCCAATGAATTGCACTAAATTCAGAAACGTGCTGGGCATATCAAGATCTATGGA
CAGCCGGCTACTTAAAGACTTTGCATTGCTTTAGAAAATGTCATAAATCTTTTTGTTGGTCGAAACCGAATCGAGTCACGCATTCT
AATGACCCGGGCAGATCCATTTAACAAGTCCCAAGTGAACTTGTTTTCAACAATACAATTTGCTTATAAATAGCATAAAATACAAT
TAAATCGTGGGTCTTGTATCATTTAAAATGTAACTGGTACAGTTCTTACTTCCTCTTTGACATTCTTTTTGGCATTTGGTGCTTGG
TAGCTTGGTAAAATATTGGGGAAAGA**GGGCAA**::::::::::::TGAAATACCAATA**TGT**TGAGTTTTTGGTCAAATTATTTCGGT
ATGCGCCAAATACATGAACGGCAAATACCTTTGTTCTTGTTGAAAGGCCTCAAA:GGAAGGAGACGAGAATAAGGGGCCACACTCC
TTCCAATGGTGTTTGAGAAATTATATCGAAAAATAAATGAAAATTCTTCCCATAATTCAAAGGCTTGTGGAAATTGCTTGGTATGT
GAAATATGTGATTTTTTTTTTTAGCTTTGCAGGAAGTTGTAAAGTTCCTCCACTTAGCTTGGTTATTATTCCATCACATTAAGCTTT
TTGGTTAATTATC

F5>
122 nt Deletion (3R:19396045-19396167)

CAGAAACGTGCTGGGCATATCAAGATCTATGGACAGCCGGCTACTTAAAGACTTTGCATTGCTTTAGAAAATGTCATAAATCTTTT
TGTTGGTCGAAACCGAATCGAGTCACGCATTCTAATGACCCGGGCAGATCCATTTAACAAGTCCCAAGTGAACTTGTTTTCAACAA
TACAATTTGCTTATAAATAGCATAAAATACAATTAAATCGTGGGTCTTGTATCATTTAAAATGTAACTGGTACAGTTCTTACTTCC
TCTTTGACATTCTTTTTGGCATTTGGTGCTTGGTAGCTTGGTAGAATATT**GGGGAAAGAGGGCAAA**::::::::::::::::::::
::
::::::::::::::::ATAAGGGGCCACACTCCTTCCAATGGTGTTTGAGAAATTATATCGAAAAATAAATGAAAATTCTTCCCAT
AATTCAAAGGCTTGTGGAAATTGCTTGGTATGTGAAATATGTGATTTTTTTTTTTAGCTTTGCAGGAAGTTGTAAAGTTCCTCCACT
TAGCTTGGTTATTATTCCATCACATTAAGCTTTTTGGTTAATTATCAACGCATACGTAAGTTTAAGTTTTTAAATCGAAGGATAAT
TCTGAGTCATCGTGAGTAAACCTCCCATCCCACGATTATTTATAAAAGGTC

G3>
22 nt deletion (3R:19396037-19396059)

ATTCAAAAACGTGCTGGGCATATCAAGATCTATGGACAGCCGGCTACTTAAAGACTTTGCATTGCTTTAAAAAATGTCATAAATCT
TTTTGTTGGTCGAAACCGAATCGAGACACGCATTCTAATGACCCGGGCAAATCCATTTAACAAGTCCCAAGTGAACTTGTTTTCAA
CAATACAATTTGCTTATAAATAGCATAAAATACAATTAAATCGGGGGTCTTGTATCATTTAAAATGTAACTGGTACAGTTCTTACT

TCCTCTTTGACATTCTTTTTGGCATTTGGTGCTTGGTAAAATATT**GGGGAAAG**::::::::::::::::::::::GCCGATTAAAT
ACCAATA**TGT**TGAGTTTTTGGTCAAATTATTCCGGTATGCGCCAAATACATGAACGGCAAATACCTTTGTTCTTGTTGAAAGGCCT
CCAAGGGAAGGAAACAAAAATAAGGGGCCACACTCCTTCCAATGGTGTTTGAAAAATTATATCGAAAAATAAATGAAAATTCTTCC
CATAATTCAAAGGCTTGTGGAAATTGCTTGGTATGTGAAATATGTGATTTTTTTTTTAGCTTTGCAGGAAGTT

"Spurious" Breaks.

Sequence of deletion-bearing lines which contain microhomology or repeated sequences (indicated in green and italics) adjacent to the 5' break site (sequence in bold). The TGT sequence is shown in red. For each sequence, the extension of deletion in nt and the molecular coordinates are provided

A3>
126 nt deletion (3R:19396087-19396213)
62 nt spurious sequence

ATTGCACTAAATTCAAAAACGTGCTGGGCATATCAAGATCTATGGACAGCCGGCTACTTAAAGACTTTGCATTGCTTTAAAAAATG
TCATAAATCTTTTTGTTGGTCGAAACCGAATCGAGACACGCATTCTAATGACCCGGGCAAATCCATTTAACAAGTCCCAAGTGAAC
TTGTTTTCAACAATACAATTTGCTTATAAATAGCATAAAATACAATTAAATCGTGGGTCTTGTATCATTTAAAATGTAACTGGTAC
AGTTCTTACTTCCTCTTTGACATTCTTTTTGGCATTTGGTGCTTGGTAAAATATT**GGGAAA*****aaagggaaggaaacaaaaataaggg***
gccaccccccttccaatggtgttgaaaaattatatca:::
:::AAAAATAAA
TGAAAATTCTTCCCATAATTCAAAGGCTTGTGGAAATTGCTTGGTATGTGAAATATGTGATTTTTTTTTTAGCTTTGCAGGAAGT

A4>
114 nt deletion (3R:19396049-19396163)
9 nt spurious sequence

TGCGGAGGAAGAGCGGAGATCCTACACCCAATGAATTGCACTAAATTCAGAAACGTGCTGGGCATATCAAGATCTATGGACAGCCG
GCTACTTAAAGACTTTGCATTGCTTTAGAAAATGTCATAAATCTTTTTGTTGGTCGAAACCGAATCGAGTCACGCATTCTAATGAC
CCGGGCAGATCCATTTAACAAGTCCCAAGTGAACTTGTTTTCAACAATACAATTTGCTTATAAATAGCATAAAATACAATTAAATC
GTGGGTCTTGTATCATTTAAAATGTAACTGGTACAGTTCTTACTTCCTCTTTGACATTCTTTTTGGCATTTGGTGCTTGGTAGCTT
GGTAGAATATTG**GGGAAAGAGG*****ataaaagac***:::
:::GAGAATAAGGGGCCACACTCCTTCCAA
TGGTGTTTGAGAAATTATATCGAAAAATAAATGAAAATTCTTCCCATAATTCAAAGGCTTGTGGAAATTGCTTGGTATGTGAAATA
TGTGATTTTTTTTTTAGCTTTGCAGGAAGTTGTAAAGTTCCTCCACTTAGCTTGGTTATTATTCCATCACATTAAGCTTTTTGGTT
AATTATCAACGCATACGTAAGTTTAAGTTTTTAAATCGAAGGATAATTCTGAGTCATCGTGAGTAAACCTCCCATCCCACGATTAT
TTATAAAAGGTCTTTGAAGCCGGCTTCAAAGATCCGCCAGCTAAAA

B5>
104 nt deletion (3R:19396052-19396156)

13 nt spurious sequence

GGAGATCCTACACCCAATGAATTGCACTAAATTCAGAAACGTGCTGGGCATATCAAGATCTATGGACAGCCGGCTACTTAAAGACT
TTGCATTGCTTTAGAAAATGTCATAAATCTTTTTGTTGGTCGAAACCGAATCGAGTCACGCATTCTAATGACCCGGGCAGATCCAT
TTAACAAGTCCCAAGTGAACTTGTTTTCAACAATACAATTTGCTTATAAATAGCATAAAATACAATTAAATCGTGGGTCTTGTATC
ATTTAAAATGTAACTGGTACAGTTCTTACTTCCTCTTTGACATTCTTTTTGGCATTTGGTGCTTGGTAGCTTGGTAGAATATT**GGG**
GAAAGAG***acaagaatattgg***::
::::::::::::::::::::::::::::::::::::::AGGAGACGAGAATAAGGGGCCACACTCCTTCCAATGGTGTTTGAGAAA
TTATATCGAAAAATAAATGAAAATTCTTCCCATAATTCAAAGGCTTGTGGAAATTGCTTGGTATGTGAAATATGTGATTTTTTTTT

C10>
11 nt deletion (3R:19396042-19396053)
2 nt spurious sequence

GGAGGAAGAGCGGAGATCCTACACCCAATGAATTGCACTAAATTCAAAAACGTGCTGGGCATATCAAGATCTATGGACAGCCGGCT
ACTTAAAGACTTTGCATTGCTTTAAAAAATGTCATAAATCTTTTTGTTGGTCGAAACCGAATCGAGTCACGCATTCTAATGACCCG
GGCAAATCCATTTAACAAGTCCCAAGTGAACTTGTTTTCAACAATACAATTTGCTTATAAATAGCATAAAATACAATTAAATCGGG
GGTCTTGTATCATTTAAAATGTAACTGGTACAGTTCTTACTTCCTCTTTGACATTCTTTTTGGCATTTGGTGCTTGGTAGCTTGGT
AAAATATT**GGGGAAAGAGGGC*****cg***:::::::::::ATGAAATACCAATA**TGT**TGAGTTTTTGGTCAAATTATTTCGGTATGCGCCAA
ATACATGAACGGCAAATACCTTTGTTCTTGTTGAAAGGCCTCAAAGGGAAGGAGACGAGAATAAGGGGCCACACTCCTTCCAATGG
TGTTTGAGAAATTATATCGAAAAATAAATGAAAATTCTTCCCATAATTCAAAGGCTTGTGGAAATTGCTTGGTATGTGAAATATGT
GATTTTTTTTTTTAGCTTTGCAGGAAGTTGTA

C11>
222 nt deletion (3R:19395943-19396165)
22 nt spurious sequence

GTGCTGGGCATATCAAGATCTATGGACAGCCGGCTACTTAAAGACTTTGCATTGCTTTAGAAAATGTCATAAATCTTTTTGTTGGT
CGAAACCGAATCGAGACACGCATTCTAATGACCCGGGCAGATCCATTTAACAAGTCCCAAGTGAACTTGTTTTCAACAATACAATT
TGCTTATAAATAGCATAAAAT***acaattaaatggaaggaaacaa***:::
::
::
:::::::GAATAAGGGGCCACACTCCTTCCAATGGTGTTTGAGAAATTATATCGAAAAATAAATGAAAATTCTTCCCATAATTCAA
AGGCTTGTGGAAATTGCTTGGTATGTGAAATATGTGATTTTTTTTTTTAGCTTTGCAGGAAGTTGTAAAGTTCCTCCACTTAGCTTG
GTTATTATTCCATCACATTAAGCTTTTTGGTTAATTATC

D5>
134 nt deletion (3R:19396086-19396220)
56 nt spurious sequence

TATCAAGATCTATGGACAGCCGGCTACTTAAAGACTTTGCATTGCTTTAGAAAATGTCATAAATCTTTTTGTTGGTCGAAACCGAA
TCGAGACACGCATTCTAATGACCCGGGCAGATCCATTTAACAAGTCCCAAGTGAACTTGTTTTCAACAATACAATTTGCTTATAAA
TAGCATAAAATACAATTAAATCGTGGGTCTTGTATCATTTAAAATGTAACTGGTACAGTTCTTACTTCCTCTTTGACATTCTTTTT

GGCATTTGGTGCTTGGTAAAATATT**GGGGAAA***caaaaataaggggcccccccccttccaagggggtttgaaaaattatatca*::::
::
::AAAAATAAATGAAAATTCTTCCCATAATTCAAAGGCTTGTGG
AAATTGCTTGGTATGTGAAATATGTGATTTTTTTTTTTAGCTTTGCAGGAAGTTGTAAAGTTCC

F2>
121 nt deletion (3R:19396046-19396167)
6 nt spurious sequence

ATGATCTGCGGAGGAAGAGCGGAGATCCTACACCCAATGAATTGCACTAAATTCAGAAACGTGCTGGGCATATCAAGATCTATGGA
CAGCCGGCTACTTAAAGACTTTGCATTGCTTTAGAAAATGTCATAAATCTTTTTGTTGGTCGAAACCGAATCGAGTCACGCATTCT
AATGACCCGGGCAGATCCATTTAACAAGTCCCAAGTGAACTTGTTTTCAACAATACAATTTGCTTATAAATAGCATAAAATACAAT
TAAATCGTGGGTCTTGTATCATTTAAAATGTAACTGGTACAGTTCTTACTTCCTCTTTGACATTCTTTTTGGCATTTGGTGCTTGG
TAGCTTGGTAGAATATT**GGGGAAAGAGGG***acgaaa*:::
::TAAGGGGCCACACTCC
TTCCAATGGTGTTTGAGAAATTATATCGAAAAATAAATGAAAATTCTTCCCATAATTCAAAGGCTTGTGGAAATTGCTTGGTATGT
GAAATATGTGATTTTTTTTTTTAGCTTTGCAGGAAGTTGTAAAGTTCCTCCACTTAGCTTGGTTATTATTCCATCACATTAAGCTTT
TTGGTTAATTATCAACGCATACGTAAGTTTAAGTTTTTAAATCGAAGGATAATTCTGAGTCATCGTGAGTAAACCTCCCATCCCAC
GATTATTTATAAAAGGTCTTTGAAGCCGGCTTCAAAGATCCGCCAGCTAAAATACCAACATCGAATTA
